# Regeneration of reptilian scales after wounding: neogenesis, regional difference, and molecular modules

**DOI:** 10.1002/reg2.9

**Published:** 2014-02-17

**Authors:** Ping Wu, Lorenzo Alibardi, Cheng‐Ming Chuong

**Affiliations:** ^1^ Department of PathologyUniversity of Southern CaliforniaLos AngelesCA90033USA; ^2^ Department of BigeaUniversity of BolognaBolognaItaly

**Keywords:** Lizard, development, molecular circuit, regeneration, wound induced neogenesis

## Abstract

Lizard skin can produce scales during embryonic development, tail regeneration, and wound healing; however, underlying molecular signaling and extracellular matrix protein expression remains unknown. We mapped cell proliferation, signaling and extracellular matrix proteins in regenerating and developing lizard scales in different body regions with different wound severity. Following lizard tail autotomy (self‐amputation), de novo scales regenerate from regenerating tail blastema. Despite topological differences between embryonic and adult scale formation, asymmetric cell proliferation produces the newly formed outer scale surface. Regionally different responses to wounding were observed; open wounds induced better scale regeneration from tail skin than trunk skin. Molecular studies suggest that neural cell adhesion molecule enriched dermal regions exhibit higher cell proliferation associated with scale growth. β‐catenin may be involved in epidermal scale differentiation. Dynamic tenascin‐C expression suggests its involvement in regeneration. We conclude that different skin regions exhibit different competence for de novo scale formation. While cellular and morphogenetic paths differ during development and regeneration of lizard scale formation, they share general proliferation patterns, epithelial−mesenchymal interactions and similar molecular modules composed of adhesion and extracellular matrix molecules.

## Introduction

Reptiles are an amniote lineage evolved from stem reptiles in the upper Carboniferous (Pough et al. 2001). A major feature enabling the success of reptiles in a terrestrial environment is the structure of their skin which provides both mechanical protection and a barrier against water loss (Maderson 1972, 1985; Alibardi 2003; Wu et al. 2004). The integument derives from progressive embryonic skin modifications during the last third of development, and is completed just before or soon after hatching (Dhouailly and Maderson 1984; Maderson 1985; Alibardi 2003, 2004; Alibardi and Toni 2008; Swadzba et al. 2009). The molecular pathways involved in reptilian skin morphogenesis (Chang et al. 2009) and regeneration are unknown.

At early stages of reptilian skin development the dermis is loose and the epidermis is flat and bi‐layered. Later the epidermis becomes undulated and forms a series of dome‐like bumps. The initial symmetric scale anlagen did not show the typical dermal condensations found in avian scutate scales and feathers (Chuong et al. 2000; Sawyer and Knapp 2003; Widelitz et al. 2003) or beneath hair placodes (Millar 2002; Botchkarev and Paus 2003). During the development of lizard scales the symmetric scale anlagen becomes slanted while suprabasal keratinocyte layers form and give rise to a partially mature corneous layer before hatching (Maderson 1985; Alibardi 1998).

When adult lizard skin is injured and a few scales are removed, a wound epidermis made of alpha‐keratin corneocytes is formed (Maderson and Roth 1972). In contrast the epidermis of large wounds (comprising more than 16 scales) can re‐form smaller and irregular scales (Maderson et al. 1978). During lizard tail regeneration, scale replacement or neogenesis occurs when new scales of similar shape and organization form in the regenerating skin (Bryant and Bellairs 1967; Alibardi 1994, 1995). From the stump that remains after tail autotomy, a mass of mesenchymal cells forms a regenerative blastema which is covered by a thick “wound epidermis.” Neogenic scales are formed in a proximal−distal gradient but with a different mechanism from that of developing scales. In fact, regenerating scales are formed from an invagination of the epidermis into the dermis, a process that grossly resembles the initial stages of hair morphogenesis (hair peg, see Bryant and Bellairs 1967). However, further microscopic studies have shown that the three‐dimensional shape of hair pegs in mammals and scale pegs in lizards is very different (Alibardi 1994, 1995).

To determine whether lizard scale regeneration and development use similar adhesion molecules and extracellular matrix proteins, we compared the expression of β‐catenin, cell adhesion molecules (neural cell adhesion molecule [NCAM] and tenascin‐C) and markers of proliferation (proliferation cell nuclear antigen [PCNA] or 5‐bromodeoxyuridine [BrdU]) during development, wound healing, and tail regeneration. We examined the expression of these marker antigens because they were previously used to characterize developmental stages of mammalian hairs, avian scales, and avian feathers (Widelitz et al. 1997). We demonstrate that in reptile skin these markers show regional differences in expression. The results show that although at the cellular level the process of regeneration differs from development, similar molecular modules are conserved.

## Results

### Regenerating tail of *Anolis carolinensis*

First, we examined scales in regenerating tails of *A. carolinensis* after autotomy. Scale regeneration was observed from day12 to day 33 at 7‐day intervals. The amputated tail formed a blastema on post wound day (PWD) 12, elongated to form a black cone at PWD 19, and became a smooth elongating tail at PWD 26 (Fig. 1A−C). No clear scales were formed at PWD 26 (Fig. 1C1, C2). By PWD 33, scales had formed along most of the length of the tail but the tip remained unscaled (Fig. 1D1, enlarged in D2). In proximal areas, short but completely mature and keeled scales were present.

**Figure 1:**
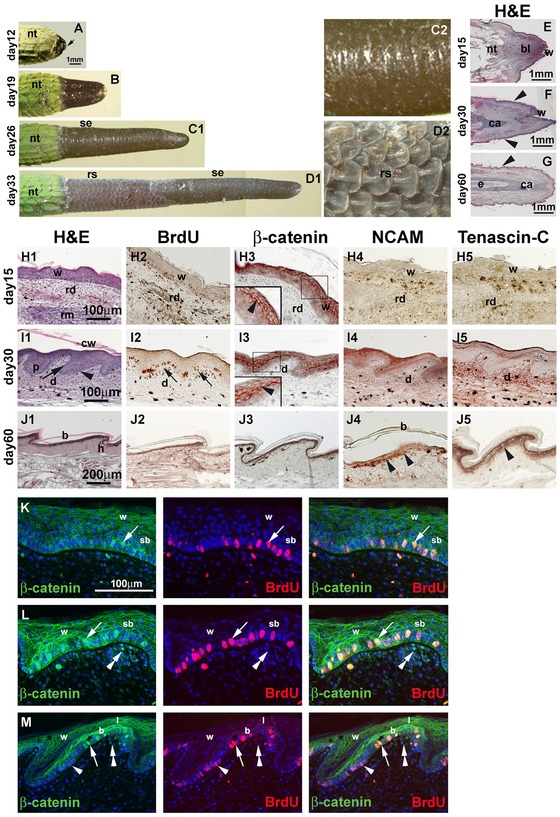
Scale neogenesis on regenerating tail of *A. carolinensis*. (A)−(D) Gross morphology of *A. carolinensis* tails at 12, 19, 26 and 33 days of regeneration. (A) Dark blastema (arrow); (B) elongating cone; (C1) regenerating tail showing the beginning of scale formation; (D1) regenerated tail with scaling in proximal regions; (C2), (D2) enlargements from the proximal part of (C1) and (D1), respectively. (E)−(J1) Skin histology with hematoxylin and eosin (H&E) stained sections: (E) blastema; (F) elongating cone (the arrowhead indicates the wound epidermis); (G) scaled tail (the arrowhead points to regenerated scales); (H1) thick wound epidermis before scaling begins; (I1) epidermal pegs in scaling epidermis; (J1) regenerated scales. (H2)−(J5) Immunocytochemistry for specific markers. (H2)−(J2) BrdU staining. (H3)−(J3) β‐catenin staining. Inserts in H3 and I3 show enlargements of the indicated areas (arrowheads indicate membranous immunostaining of keratinocytes). (H4)−(J4) NCAM staining (arrowheads indicate more intense staining). (H5)−(J5) Tenascin‐C staining (the arrowhead indicates a more intensely labeled region). (K)−(M) Confocal immunocytochemistry of day 30 regenerating skin. Left panel, β‐catenin staining. Middle panel, BrdU staining. Right panel, combined. (K) Double‐labeled nuclei (arrow) in the undulated wound epidermis with diffuse β‐catenin labeling in suprabasal layers. (L) Early peg showing double‐labeled nuclei (arrow) and β‐catenin in suprabasal keratinocytes. (M) Elongated peg with double‐labeled nuclei (arrow) and BrdU single‐labeled nuclei (arrowhead). Double arrowheads in (L) and (M) indicate the β‐catenin nuclear positive cells in the mesenchyme. b, beta‐layer; bl, blastema; ca, regenerating cartilage; d, dermis; e, ependyma; h, hinge; l, lacunar epithelium; p, epidermal peg; nt, normal tail; rd, regenerated dermis; rm, regenerated muscle; rs, regenerated scale; sb, suprabasal layer; se, scaling epidermis; w, wound epidermis.

Scale regeneration (neogenesis) was studied histologically at different time points during tail regeneration (Fig. 1E−G). The elongating cone at PWD 15 (Fig. 1E) showed a thick wound epidermis (Fig. 1H1). In the elongating tail at PWD 30 (Fig. 1F) numerous epidermal pegs were present (Fig. 1I1). In fully regenerated PWD 60 tails (Fig. 1G) both dermis and epidermis appeared normal (Fig. 1J1). We compared cell proliferation (BrdU staining) as well as expression patterns of β‐catenin, NCAM and tenascin‐C at different regeneration time points.

Numerous basal epidermal cells but only few mesenchymal cells were BrdU positive in the thick PWD 15 wound epidermis (Fig. 1H2). β‐catenin was localized in the cell membrane of suprabasal keratinocyte, along the outlines of lacunar and presumptive clear cells (Fig. 1H3 and inset, arrowhead). NCAM and tenascin‐C immunostaining showed little or no reactivity at this stage (Fig. 1H4, 5).

In elongating tails at PWD 30, BrdU‐positive cells mainly localized within the longest side of the pegs, where the outer scale surface was forming (Fig. 1I2, arrows). β‐catenin localized mainly in the pegs, along the perimeter of the wound epidermis, in lacunar cells and in the cytoplasm of presumptive beta‐cell (Fig. 1I3 and the inset, arrowhead). This layer formed a line of intensely labeled squamous cells marking the origin of the outer surface of neogenic scales. NCAM immunostaining was prevalent in the wound epidermis, outlining lacunar cells in the epidermal pegs and especially in the forming beta‐cell layer (Fig. 1I4). Very little NCAM staining was present in the mesenchyme. Tenascin‐C was expressed especially in the forming dense dermis localized at the base of neogenic scales (Fig. 1I5).

In regenerated tails at PWD 60, few BrdU‐labeled epidermal cells were seen in the scales (Fig. 1J2) while β‐catenin staining remained only in living epidermal keratinocytes (Fig. 1J3). The NCAM staining in the epidermis was weak but it was more intense in the dermis close to the epidermal−dermal junction of the outer scale surface (Fig. 1J4). Tenascin‐C localized to the basal epidermis and contacted dermal cells along the entire neogenic scales (Fig. 1J5).

To further examine the relation between cell proliferation and β‐catenin expression in regenerating scales, we performed confocal microscopic analysis of BrdU (red color) and β‐catenin (green color) at PWD 30. These samples included slightly different stages of regenerating scales, and were located from proximal to distal regions along the regenerating tail but not in apical regions (Fig. 1K−M). Most proliferating nuclei in the elongating distal peg epidermis were double labeled (proliferation and activated toward differentiation) for BrdU and nuclear β‐catenin (Fig. 1K−M; arrows). Only nuclei in the lowermost part of the peg were single labeled for BrdU (Fig. 1M, arrowhead). Single‐labeled β‐catenin positive nuclei were located toward the apical dermis of the forming scale (Fig. 1L, M, double arrowhead). β‐catenin immunofluorescence was also seen in the wound epidermis cytoplasm and was stronger in the differentiating beta‐cell layer.

### Wound healing and scalation of the skin in normal tail of *A. carolinensis*

In mice, small wounds can induce the formation of scars which cannot form hairs or glands. In contrast, large wounds (1 cm × 1 cm) on the backs of mice were found to induce hair neogenesis (Ito et al. 2007). African spiny mice (Acomys), whose skin also undergoes autotomy, can regenerate skin with all appendages and dermis (Seifert et al. 2012). We wondered whether scales could regenerate when a skin wound is produced on the tail, rather than removal of tail by autotomy. In the normal *A. carolinensis* tail, scales are overlapping with a hinge region present in the middle. A 2 × 5 mm skin region was surgically removed from a normal tail. After 1 week, a smooth, variably dark wound epidermis was formed and scales become visible by PWD 28 (Fig. 2A1−A3). An increased number of small, round or irregular scales were seen by PWD 45 (Fig. 2B1−B3). The lack of a changing pigmentation in the regenerated skin suggests that the epidermal−dermal chromatophoric unit was not present.

**Figure 2:**
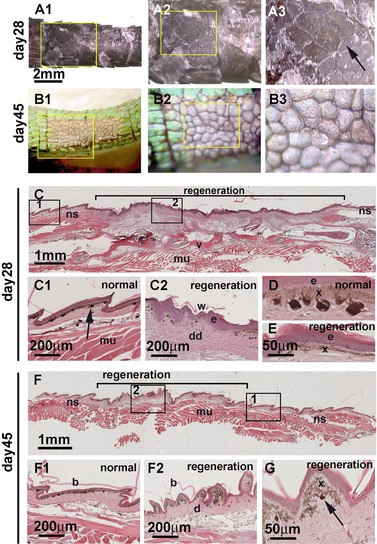
Scale regeneration in wounded *A. carolinensis* tail. (A1)−(B3) Gross morphological view of wounded *A. carolinensis* tail after 28 and 45 days. (A1) 28 days post‐wounding, scales are present on the dark skin (enlarged in A2 and A3). (B1) 45 days post‐wounding, scaling is almost complete but neither shape nor color matches those of normal scales (enlarged in B2 and B3). (C)−(E) Histological images (H&E) of the repaired skin highlighting the chromatophore organization. (C) Regenerated skin present between normal scales. Boxed areas are enlarged to show details of normal scales (C1, the arrow indicates melanophores) and regenerated skin (C2). The regenerated epidermis rests on a dense dermis containing few melanophores. (D) Detail of a normal chromatophoric unit with melanophore elongation directed toward the epidermis. (E) Detail of regenerated skin showing absence of chromatophore organization and lack of melanophores. (F) At 45 days after wounding the skin shows small irregular scales (details in squares 1 and 2): (F1) normal scale; (F2) irregular regenerated scales showing sparse chromatophores in the dense dermis. (G) Detail of regenerated skin showing few melanophores (arrow) present underneath the xanthophore layer (compared with normal scale in D). b, beta‐layer; d, dermis; dd, dense dermis; e, epidermis; mu, muscles; ns, normal scales; v, vertebral bone; w, wound epithelium; x, xanthophore layer.

The histological examination of the healed skin showed that wound epithelium lying between the normal scaled regions (ns) was thickened and formed small, round structures in comparison to normal scales at PWD 28 (Fig. 2C, C2). Ordered scales had not yet formed. The multilayered wound epidermis formed a corneous (alpha‐) layer but not a distinct and continuous beta‐layer. In normal scales, melanophores of the dermal chromatophoric unit sent projections toward the upper iridophores and xanthophores (Fig. 2D). In the wounded skin, melanophores of the homogeneously dense dermis were randomly scattered along the base of the epidermis and often only a layer of xanthophores was seen (Fig. 2E).

At PWD 45 some irregular and short scales regenerated (Fig. 2F). These scales resembled tuberculate scales (Fig. 2F2). The mature scales showed a beta‐layer and their dermis appeared uniformly dense with no subdivisions. As opposed to normal scales (Fig. 2F1), few dark melanophores lacking projections toward the epidermis were seen in the deep dermis while a layer of xanthophores (and probably ìridophores) was present beneath the epidermis (Fig. 2G). These chromatophores, however, did not appear organized in a chromatophoric unit.

### Wound healing and scalation of the skin in the body of *A. carolinensis*

To test whether body scales can also regenerate as in tail scales, we surgically removed ∼25 mm^2^ body skin at the dorsal−ventral junction between the forelimb and hindlimb. The healing process generated a scarred, wrinkled, and poorly pigmented skin at PWD 28 (Fig. 3A1−A3, arrow). At PWD 45 no clearly defined scales were formed and the healing skin appeared grey‐brown in color (Fig. 3B1−B3, arrow).

**Figure 3:**
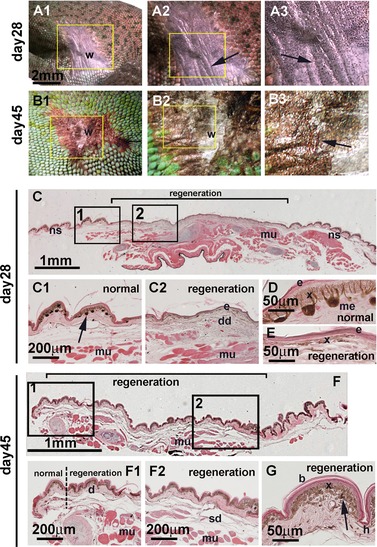
Scale regeneration in wounded *A. carolinensis* body. (A1)−(B3) Gross morphological view of repaired skin 28 days and 45 days after wounding with different magnifications: (A1) little scalation is visible and the color appears grey and unpatterned; (B1) this neogenic skin has neither normal color nor pattern. Arrows in A2, A3 and B3 indicate skin furrows. (C), (D) Histological aspects (H&E) at 28 days. (C) Regenerated skin located between normal scales. The boxed areas indicate some regions analyzed at higher magnification. (C1) Normal scales; (C2) undulated surface of the regenerated epidermis resting upon a dense dermis where no distinct scales are formed. (D) The dermal chromatophoric unit in a normal scale. (E) The dermis with xanthophores and flat melanophores indicating the absence of a chromatographic unit. (F), (G) Histological aspects (H&E) at 45 days. (F) Wounded skin with numerous folds resembling scales. The shape of these scales is irregular and incomplete. The boxes show higher magnified details. The left side of (F1) shows normal, unwounded skin. The right side of (F1) and all (F2) are the regenerated wound regions, and they show irregular tuberculate‐like scales. (G) Detail on a tuberculate scale showing the thick layer of irregularly distributed xanthophores and melanophores, as indicated by the arrow. b, beta‐layer; d, dermis; dd, dense dermis; e, epidermis; h, hinge; mu, muscles; ns, normal scales; sd, superficial dermis; w, wound epithelium; x, xanthophore layer.

The histological analysis at PWD 28 showed that, compared with the small normal scales present in other body regions (Fig. 3C, C1), the wounded skin comprised a thin, flat epidermis covered by a narrow corneous layer (Fig. 3C2). Normal scales in unwounded skin showed an ordered chromatophore distribution, forming a dermal chromatophoric unit where melanophore elongation passed through iridophore and xanthophore layers to reach the epidermis (Fig. 3D). In the wound bed, small grooves irregularly folded the epidermis into hinge‐like regions but clearly defined scales were not present (Fig. 3E). Few melanophores were seen in the dense dermis that contained sparse xanthophores but no stratified chromatophoric units were present (Fig. 3E). At PWD 45 some regenerated scales appeared irregular in shape and no overlapping scales were observed (Fig. 3F, enlarged in F1 and F2). Sparse melanocytes colonized this epidermis and rare dermal chromatophores were present above or among the xanthophores. Therefore a chromatophoric unit was absent in this healed skin of the tail and body regions in *A. carolinensis* (Fig. 3G). We conclude that wounded *A. carolinensis* tail and body skin only regenerated small, irregular scales with no chromatographic units. Wounds on the tail skin (5 mm × 2 mm) induced a larger regenerative response than wounds (5 mm × 5 mm) on the body skin. It is possible that a wound with a similar size might produce an even better healing response in the tail.

### Wound healing and scalation of the skin in the normal *Iguana iguana* tail

Irregular scale formation in normal *A. carolinensis* tails prompted us to ask whether scales could re‐form in the absence of tail regeneration. This was tested in the green iguana (*I. iguana*) whose tail does not generally regenerate or forms in some cases a short and clubbed tail (Alibardi 2010). An ∼25 mm^2^ tail skin wound induced a dark healing skin at PWD 21 (Fig. 4A). Irregularly shaped scales appeared at PWD 49 (Fig. 4C) and PWD 80 (Fig. 4E).

**Figure 4:**
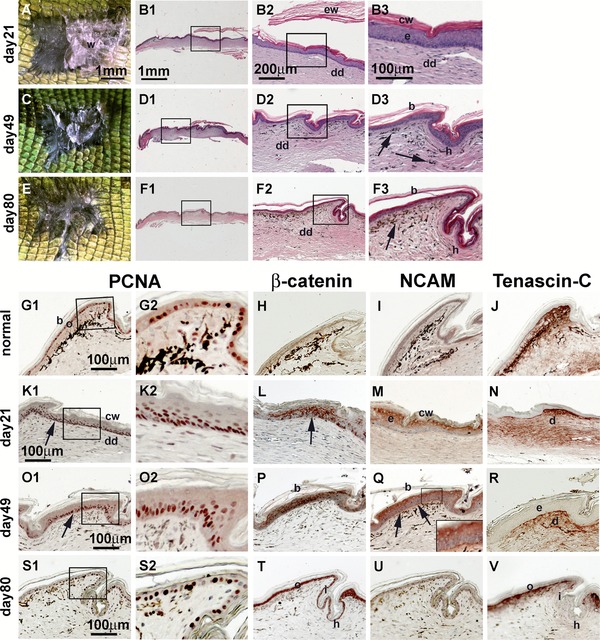
Regeneration of scales on the open wounds on *I. Iguana* tail skin. (A), (C), (E) Macroscopic features of scaling. (B1)−(B3), (D1)−(D3), (F1)−(F3) H&E staining with different magnification. (A)−(B3) 21 days after wound. (C)−(D3) 49 days. (E)−(F3) 80 days. Arrows in (D3) and (F3) indicate melanophores in dense dermis. (G1)−(V) Immunocytochemical distribution of different marker proteins in normal and regenerated scales at 21, 49 and 80 days post‐wounding: (G1), (K1), (O1), (S1) PCNA staining; (G2), (K2), (O2), (S2) higher magnification images. Arrows in (K1), (O1) indicate that PCNA‐positive cells are present in multiple regenerating scale layers. (H), (L), (P), (T) β‐catenin staining. Arrow in (L) indicates a broad distribution of β‐catenin at day 21. (I), (M), (Q), (U) NCAM staining. Arrows in (Q) indicate the presence of NCAM at the epidermal−dermal junction in regenerating scales. Inset in (Q) is the higher magnification. (J), (N), (R), (V) tenascin‐C staining. b, beta‐layer; cw, corneous layer of the wound epidermis; dd, dense dermis; e, epidermis; ew, exfoliating wound epidermis; h, hinge region; i, inner scale surface; o, outer scale surface; w, wound epidermis.

Histological examination at PWD 21 showed a relatively thick, wound epidermis forming a multilayered stratum corneum (Fig. 4B1−B3). At PWD 49 the skin appeared irregularly scaled and different length scales appeared with random orientation (Fig. 4D1−D3). The epidermis comprised cornified beta‐ and alpha‐layers but the thick dermis was uniform and contained sparse melanophores (Fig. 4D3, arrows). At PWD 80, numerous collagen bundles formed irregularly oriented eosinophilic fibres in the deep dense dermis (Fig. 4F1−F3). The regenerated scales show a thick beta‐layer and numerous dermal melanophores (Fig. 4F3, arrow).

Normal, post‐shedding scales had PCNA‐positive basal epithelial cells (Fig. 4G1, enlarged in G2). β‐catenin and NCAM were faintly expressed in the epidermis and dermis, respectively (Fig. 4H, I). Tenascin‐C immunoreactivity was mainly observed in the core dermis located underneath the basal epidermis of the outer scale surface and among the collagen bundles of the dense laminar dermis (Fig. 4J).

At PWD 21 numerous PCNA‐positive cells were in the wound epidermis but few were in the underlying superficial dermis (Fig. 4K1, arrow; enlarged in K2). β‐catenin was present in sparse basal and suprabasal cell cytoplasm but was absent in corneous wound epithelium (Fig. 4L, arrow). NCAM was only seen in suprabasal and pre‐corneous epidermal cells (Fig. 4M). Dermal tenascin‐C localized beneath the outer scale surface of both normal and regenerated scales. A higher tenascin‐C concentration was seen in elevated skin regions (Fig. 4N).

At PWD 49, PCNA‐positive cells were mainly seen in the basal epidermis and in sparse dermal cells (Fig. 4O1, arrow; enlarged in O2). β‐catenin was prevalent along the perimeter of suprabasal and pre‐corneous epithelial cells and in sparse dermal cell nuclei (Fig. 4P). NCAM was present in the epidermis but appeared more intense in pre‐corneous (alpha‐) cells. A thin immune‐positive layer marked the epidermal−dermal junction of this scale forming epidermis (Fig. 4Q, arrows; enlarged in inset). Tenascin‐C was localized particularly in the dermis present underneath the outer scale surface (Fig. 4R).

At PWD 80 fewer epithelial nuclei along the outer and inner scale surfaces were PCNA positive (Fig. 4S1, enlarged in S2). β‐catenin was present in the epidermis of the outer, inner and hinge scale region (Fig. 4T). NCAM labeling appeared low to completely absent in scales (Fig. 4U). Tenascin‐C was present in the superficial dermis beneath the basal epidermis of the outer scale surface and appeared to be absent from the inner surface and hinge regions (Fig. 4V). Our data suggest that *I. iguana* skin regeneration in the tail forms irregularly shaped scales. Over time, normal molecular expression patterns were gradually restored in irregularly shaped regenerating scales.

### Scale development in *Podarcis muralis* embryos

How does adult scale regeneration differ from embryonic scale development? To examine lizard scale development, we prepared sections from *P. muralis* embryos. *P. muralis* instead of *A. carolinensis* has been used here because the embryos are larger and more accessible. Besides, scale development is similar in all lizards studied so far, and their morphogenesis is particularly known in *P. muralis* (Dhouailly and Maderson 1984; Alibardi 1998). During embryogenesis, the lizard skin changed from an initially loose dermis with a flat and bi‐layered epidermis into an undulated epidermis which formed a series of dome‐like bumps of variable extension in a body region dependent fashion (Fig. 5A, B). The initial symmetric embryonic stage (ES) 32−33 scale anlagen showed only a loose mesenchyme beneath one or two epidermal layers and a flat periderm (Fig. 5B). At ES 34−35, the symmetric scale anlagen became slanted while suprabasal keratinocytes were generated beneath the external and flat periderm (Fig. 5C). At ES 38 many differentiating epidermal layers formed mainly on the outer scale surface (clear, oberhautchen and pre‐beta‐layer) while the periderm cornified (Fig. 5D).

**Figure 5:**
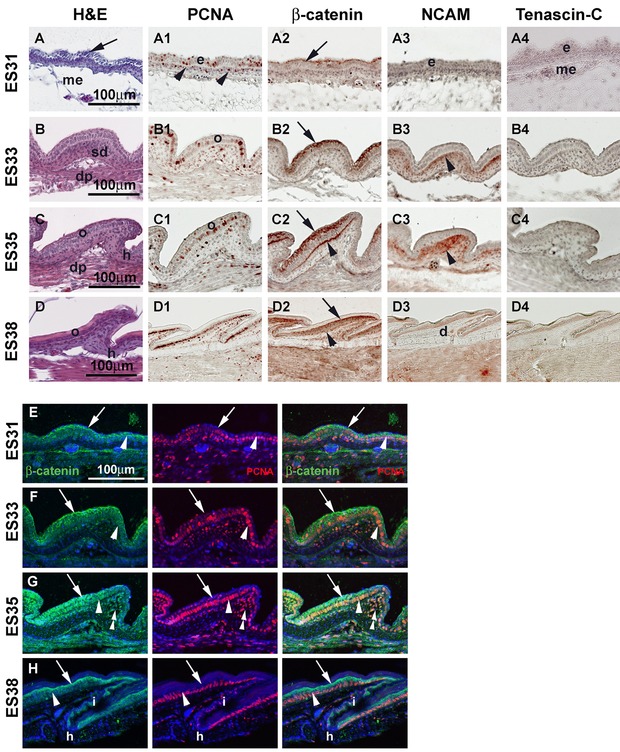
Development of scales in *P. muralis* embryos. (A)−(A4) Developing ventral scales at embryonic stage 31. (B)−(B4) Stage 33. (C)−(C4) Stage 35. (D)−(D4) Stage 38. (A)−(D) H&E staining. Arrow in (A) indicates the periderm. (A1)−(D1) PCNA staining. Arrowheads in (A1) indicate PCNA‐labeled mesenchymal cells. (A2)−(B2) β‐catenin staining. β‐catenin localized in the periderm (arrows in A2−C2) later is present in the differentiating epidermis (arrow in D2) and epidermal−dermal junction (arrowheads in C2 and D2). (A3)−(D3) NCAM staining. Arrowheads in (B3), (C3) indicate the expression of NCAM in the mesenchyme present underneath the elongating outer scale surface. (A4)−(D4) Weak immunoreactivity for tenascin‐C at all stages. (E)−(H) Confocal images of progressively developing scales with immunostaining for PCNA (red, arrowhead), β‐catenin (green, arrows) and DAPI (blue). Left panel, β‐catenin staining. Middle panel, PCNA staining. Right panel, combined. Double arrowheads in (G) indicate the double β‐catenin and PCNA nuclear positive cells in the mesenchyme. d, dermis; dp, deep dermis; e, epidermis; h, hinge region; i, inner scale surface; me, mesenchyme; o, outer scale surface; sd, superficial dermis.

At ES 31 the epidermis of the ventral skin showed a slightly waved shape. PCNA‐positive cells were randomly distributed in the epidermis and less frequently in the dermis (Fig. 5A1). β‐catenin localized in randomly sparse nuclei of the periderm, epidermis, and dermis (Fig. 5A2). NCAM was weakly present in the periderm and in basal epidermal cells (Fig. 5A3). Tenascin‐C was not detected in either epidermis or dermis at any stage (Fig. 5A4−D4). At ES 33 the epidermis formed symmetric or slightly asymmetric scales. PCNA‐labeled cells were randomly distributed mainly within the epidermis. Some periderm cells were also labeled (Fig. 5B1). β‐catenin appeared in the outer epidermal layer of the outer scale surface and in the periderm (Fig. 5B2, arrow). NCAM was present in the scale dermis, especially beneath the forming outer scale surface (Fig. 5B3). In asymmetric scales at ES 35, PCNA‐positive cells were seen in the elongating outer scale surface epidermis and in sparse mesenchymal cells (Fig. 5C1). β‐catenin immunoreactivity was only seen in the periderm, in the following oberhautchen layer (Fig. 5B2, arrow), and in the dermis (arrowhead) beneath the outer scale surface (Fig. 5C2). NCAM was mainly detected in the dermis located underneath the outer scale surface (Fig. 5C3). At ES 38, few PCNA‐labeled cells were seen in the basal elongated outer scale epidermis (Fig. 5D1) while β‐catenin showed a weak but similar expression pattern as at ES 35 (Fig. 5D2). NCAM immunoreactivity was absent in keratinized scales and dermis (Fig. 5D3). We also observed staining in scales from the dorsal region. They showed a similar expression pattern as that found in ventral scales.

We used confocal microscopy to examine β‐catenin (green) and PCNA (red) expression in developing scales. β‐catenin was present in the epidermis, especially in the periderm at ES 31 (arrow), and numerous other epidermal and few dermal cells were positive for PCNA (arrowhead) (Fig. 5E). By ES 33, scales have become asymmetric. β‐catenin was still present throughout the epidermis but it concentrated in the outer surface (arrow) whereas numerous PCNA‐positive cells were also seen in the inner surface (arrowhead) (Fig. 5F). At ES 35, β‐catenin was present in differentiating epidermal cells of the elongating outer scale surface, especially those of the differentiating beta‐layer (Fig. 5G, arrow). Numerous basal epidermis cells in the outer scale surface were double stained for nuclear β‐catenin and PCNA (Fig. 5G, arrowhead). In the dermis, PCNA−β‐catenin double‐labeled cells were particularly concentrated toward the scale tip (Fig. 5G, double arrowhead). At ES 38, PCNA‐positive cells remained localized in the basal layer of the elongated outer scale surface (Fig. 5H, arrow). β‐catenin remained in suprabasal and differentiating keratinocytes of the alpha‐layer (arrow) but disappeared in the cornified beta‐ and alpha‐layers (Fig. 5H). Nuclear β‐catenin or double‐labeled cells for β‐catenin and PCNA were absent at this late stage of scale morphogenesis.

## Discussion

### Localization of different markers during scale regeneration and development

Regeneration of the lizard tail and skin has garnered much attention as wound healing models lately because the healing occurs without forming scars (McLean and Vickaryous 2011; Delorme et al. 2012). The present study shows that embryonic scale development (Fig. 6A−D) and adult scale regeneration (Fig. 6E−H) follow similar proliferative, signaling, and dermal expression patterns. In the embryo, scale development begins with a narrow epidermis and a periderm resting on the mesenchymal dermis. In contrast, during regeneration a multilayered wound epidermis in the regenerating tail forms a corneous layer before pegs invaginate.

**Figure 6:**
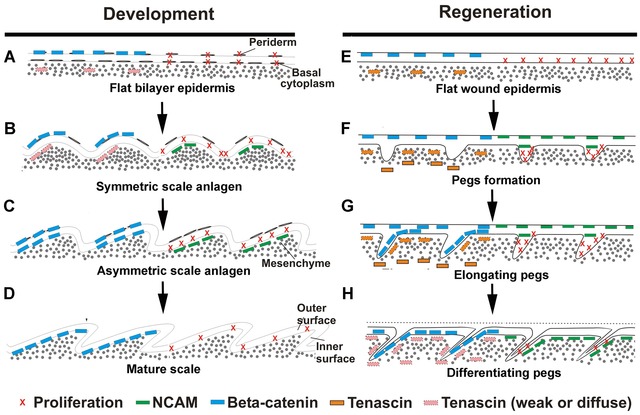
Comparison of scale development in reptilian embryo, scale regeneration in adult reptiles, and hair follicle neogenesis in mammals. (A)−(D) Embryonic scale development from flat bilayer epidermis to symmetric scale anlagen to asymmetric scale anlagen and further to mature scales. (E)−(H) Scale regeneration from flat wound epidermis to peg formation to elongating pegs and further to differentiating pegs. Cell proliferation and molecular expression are shown. For each panel, expression patterns for β‐catenin and tenascin‐C are shown in the left two scale primordia while proliferation and NCAM are shown in the right two primordia.

Cell proliferation, determined using either PCNA or BrdU labeling in both developing and regenerating scales, shows a higher concentration of proliferating cells in the outer scale surface. This is mainly localized in the germinal epidermis that forms the outer scale surface (Alibardi 1998, 2004) and corresponds to the distal layer of the peg epidermis of early regenerating scales (Alibardi 1994, 1995).

In both developing and regenerating scales, mesenchymal NCAM expression may be associated with proliferation and expansion of the basal elongating outer scale surface (Alibardi 1995, 2004). This pattern differs from that of elongating avian scales where the inner scale surface and hinge regions express the highest NCAM (Shames et al. 1991; Sawyer and Knapp 2003). Therefore it appears that the morphogenesis of lizard and chick scales occurs through different mechanisms.

The relative position of NCAM in the mesenchyme to highly proliferating epithelial regions suggests that there may be cross‐talk between these two cell populations. These regions are localized in different areas of reptilian scales, avian scales, feathers, and hairs (Shames et al. 1991; Widelitz et al. 1997, 2003). However, it should be noted that areas of intense epithelial−mesenchymal communication are characterized by a fenestrated or discontinuous basement membrane (Alibardi 2004). NCAM is absent in the deeper and denser dermis of normal and regenerating scales.

β‐catenin has a prevalent nuclear localization in epidermal cells during early stages of development (waved epidermis) or regeneration (pegs formation). Later nuclear β‐catenin is mainly present in differentiating keratinocytes of the beta‐layers but not of the alpha‐layers formed underneath (Fig. 5H). This suggests that the Wnt‐pathway activation may induce the differentiation in particular of the beta‐cells.

### Wound healing and regeneration

Two months after wounding, following an initial phase of re‐epithelialization with the formation of a thick wound epidermis and dermis, scales of irregular shape and size eventually form. While the complete scale morphogenesis and differentiation occur in the blastema of the regenerating tail, this is not the case when we produce large full thickness wounds, surrounded by old scales, on both the tail and trunk skin. In lizards which are capable (*A. carolinensis*) or poor (*I. iguana*) in tail regeneration, the new scales are irregular and smaller, and do not match the even pattern present in the surrounding normal scales in either shape or pigmentation. In fact, repaired skin dermis does not reform the dermal chromatophoric unit, and in particular lacks melanophores and probably iridophores so that it is not capable of a physiological color change (Taylor and Hadley 1970).

PCNA was distributed uniformly throughout the entire epidermis suggesting that most of the irregular scales do not have distinguishable outer and inner surfaces. These irregularly shaped scales may be folds in the skin surface. In development and scale neogenesis during tail regeneration, NCAM‐positive mesenchyme is in contact with the elongating outer scale surface. In contrast, NCAM is absent in the evenly dense dermis formed in wounded scales of *I. iguana* tails. This finding suggests that a dense dermis corresponding to the deep dermis of normal‐regenerating scales form in this skin. The localization of NCAM in the initial wound epidermis (Fig. 4M) and scaling epidermis (Fig. 4Q) is a unique case observed only in *I. iguana*. The significance of this different localization remains unexplained.

In some reptiles, the dermis is organized in dermal chromatophoric units. Following wound healing, chromatophoric dermal units are not re‐formed. The present study shows that the complete restoration of the dermal chromatophoric unit only occurs in the neogenic scales of the regenerated tail. Thus the new tail re‐establishes its micro environment including factors that are required for proper pigmentation.

### Adult regeneration and embryonic development can follow different morphogenetic paths to establish the same scale morphology

As in previous studies, also the present study suggests that the morphogenetic mechanism of scale regeneration and development in lizards is apparently different (Table 1). In development scales form by evagination while during regeneration scales form by invagination. However, the final scale morphology is similar. The examination of the tissue distribution of molecules expressed during these morphogenetic events shows similar patterns of proliferation, epithelial−mesenchymal interactions and signaling expression. Regions of intense epithelial−mesenchymal interactions during scale morphogenesis show high levels of NCAM and cell proliferation. Tenascin‐C accompanies the extension of the outer scale surface while β‐catenin and probably the Wnt signaling pathway are involved in keratinocyte differentiation, especially of the beta‐layer. These findings suggest that different molecular modules, composed of similar molecular circuits, can be called upon to the reach the same final morphology.

**Table 1:** Summary of lizard scale regeneration and embryonic scale development

	Scales in skin
Adult or juvenile	
*A. carolinensis* tail autotomy	Scales are fully regenerated in regenerated tails. Neogenic scales are formed through invagination
*A. carolinensis* tail skin wound	Scales are tiny and abnormal in shape with increased numbers
*A. carolinensis* tail body wound	Scales are abnormal in shape
*I. iguana* tail skin wound	Scales are abnormal in shape
Embryo	
*P. muralis* embryos	Scales develop through evagination

## Materials and methods

### Experiments and animals

Embryos of *P. muralis* were obtained from eggs previously collected in the field (Alibardi 1998) and analyzed at ES 32 (*n* = 2), 33 (*n* = 2), 35 (*n* = 2), and 38 (*n* = 2) according to Dufaure and Hubert (1961). Tissues (2−3 mm) collected from the dorsum and belly were fixed in 4% paraformaldehyde in 0.1 mol/L phosphate buffer for 5−6 h, rinsed in buffer, dehydrated in ethanol, xylene and embedded in paraffin. We have utilized *P. muralis* embryos to study skin morphogenesis because they are larger and experimentally more accessible than *A. carolinensis* embryos.

Adult green anole (*A. carolinensis*) (1 year old) and juvenile green iguanas (*I. iguana*) (1 year old) came from local vendors. Animals were kept at the USC's animal facility maintaining day (28°C) and night (23°C) temperatures, respectively. The photoperiod consisted of 10 h of light and 14 h of dark, respectively. Green anoles were fed daily with mealworms and with crickets every week. Green iguanas were fed with green vegetables and Natural Adult Iguana Food Pellets (Zoo Med, Costa Mesa, CA, USA). All procedures were approved by the USC Institutional Animal Care and Use Committee.

*A. carolinensis* tails were broken by autotomy, holding the external tip or pulling the tail region causing breakage at vertebral fracture planes (*n* = 10). For skin biopsies, animals were anesthetized by an intramuscular injection of ketamine (50 mg/kg) and xylazine (5 mg/kg). The area for biopsies was initially drawn with a pen and this line was traced with a scalpel to about 1 mm in depth. The skin was excised by lifting it out with forceps and removing it with a scalpel. We tried our best to avoid damaging the underlying structures (muscles, vertebrae). For *A. carolinensis*, ∼25 mm^2^ (5 mm × 5 mm) skin was removed from the body (lateral side at the dorsal−ventral junction, and between the forelimb and hindlimb). A piece of skin ∼10 mm^2^ (5 mm in length × 2 mm in width) was removed from the tail region to accommodate the size of a tail. Both tail and body biopsies were collected from the left side at the same time (*n* = 4). For *I. iguana*, ∼25 mm^2^ (5 mm × 5 mm) skin was removed from the tail (*n* = 6). After surgery, animals were treated with analgesia (ketoprofen, 2 mg/kg) daily for 3 days. Animals were euthanized by 3 months.

For pulse labeling, the animals were injected with BrdU (Sigma) intraperitoneally (50 mg/kg). After 3 h the animals were euthanized and samples were collected.

### Microscopic methods

Adult samples were fixed in 4% paraformaldehyde in 0.1 mol/L phosphate buffer overnight and decalcified with 0.5 mol/L ethylenediaminetetraacetic acid for 3 days to 1 week at 4°C. Seven micron paraffin sections were prepared. Sections were treated for antigen retrieval with 0.01 mol/L citrate buffer (pH 6.0) by microwaving for 6 min. Immunohistochemistry was performed according to Jiang et al. (1998) using the following antibodies (1:200 dilution): β‐catenin (Sigma; C2206, St Louis, MO); PCNA (Chemicon; CBL407, Billerica, MA); NCAM and tenascin‐C (Chuong and Chen 1991). Secondary antibodies were either biotinylated anti‐mouse IgG or anti‐rabbit IgG (Vector Laboratories, Burlingame, CA, USA; 1:200 dilution). The tertiary antibody was streptavidin (Vector Laboratories, 1:200 dilution). An AEC substrate kit (Vector Laboratories) was used to develop the staining. Hematoxylin was used to perform faint counterstaining. BrdU staining was performed according to Wu et al. (2004). For BrdU (BD; 347580; 1:200 dilution)/β‐catenin or PCNA/β‐catenin double staining, secondary antibodies Alexa Fluor anti‐rabbit‐488 (A11008) and anti‐mouse‐546 (A11030) from Invitrogen (Grand Island, NY) were used at a 1:200 dilution. 4’,6‐Diamidino‐2‐phenylindole (DAPI) was used to visualize the nuclei. Stained sections were imaged with a Zeiss 510 confocal microscope.
